# Continuous Monitoring of Tau-Induced Neurotoxicity in Patient-Derived iPSC-Neurons

**DOI:** 10.1523/JNEUROSCI.2590-20.2021

**Published:** 2021-05-12

**Authors:** Derek H. Oakley, Naomi Klickstein, Caitlin Commins, Mirra Chung, Simon Dujardin, Rachel E. Bennett, Bradley T. Hyman, Matthew P. Frosch

**Affiliations:** ^1^Harvard Medical School, Boston, Massachusetts 02115; ^2^Department of Neurology, Massachusetts General Hospital, Boston, Massachusetts 02114-2696; ^3^Department of Pathology, Massachusetts General Hospital, Boston, Massachusetts 02114-2696; ^4^C.S. Kubik Laboratory for Neuropathology, Massachusetts General Hospital, Boston, Massachusetts 02114; ^5^Massachusetts Alzheimer's Disease Research Center, Charlestown, Massachusetts 02129

**Keywords:** iPSC, MAPT, Alzheimer's disease, FTLD, tau seeding, tau aggregation, neurotoxicity, PSEN1 L435F

## Abstract

Tau aggregation within neurons is a critical feature of Alzheimer's disease (AD) and related tauopathies. It is believed that soluble pathologic tau species seed the formation of tau aggregates in a prion-like manner and propagate through connected neurons during the progression of disease. Both soluble and aggregated forms of tau are thought to have neurotoxic properties. In addition, different strains of misfolded tau may cause differential neurotoxicity. In this work, we present an accelerated human neuronal model of tau-induced neurotoxicity that incorporates both soluble tau species and tau aggregation. Using patient-derived induced pluripotent stem cell (iPSC) neurons expressing a tau aggregation biosensor, we develop a cell culture system that allows continuous assessment of both induced tau aggregation and neuronal viability at single-cell resolution for periods of >1 week. We show that exogenous tau “seed” uptake, as measured by tau repeat domain (TauRD) reporter aggregation, increases the risk for subsequent neuronal death *in vitro*. These results are the first to directly visualize neuronal TauRD aggregation and subsequent cell death in single human iPSC neurons. Specific morphologic strains or patterns of TauRD aggregation are then identified and associated with differing neurotoxicity. Furthermore, we demonstrate that familial AD iPSC neurons expressing the PSEN1 L435F mutation exhibit accelerated TauRD aggregation kinetics and a tau strain propagation bias when compared with control iPSC neurons.

**SIGNIFICANCE STATEMENT** Neuronal intracellular aggregation of the microtubule binding protein tau occurs in Alzheimer's disease and related neurodegenerative tauopathies. Tau aggregates are believed to spread from neuron to neuron via prion-like misfolded tau seeds. Our work develops a human neuronal live-imaging system to visualize seeded tau aggregation and tau-induced neurotoxicity within single neurons. Using an aggregation-sensing tau reporter, we find that neuronal uptake and propagation of tau seeds reduces subsequent survival. In addition, human induced pluripotent stem cell (iPSC) neurons carrying an Alzheimer's disease-causing mutation in presenilin-1 undergo tau seeding more rapidly than control iPSC neurons. However, they do not show subsequent differences in neuronal survival. Finally, specific morphologies of tau aggregates are associated with increased neurotoxicity.

## Introduction

Pathologic phosphorylation and subsequent aggregation of the microtubule binding protein tau (MAPT) is believed to play a central role in driving cognitive decline in Alzheimer's disease (AD) and related tauopathies. In these diseases, hyperphosphorylated tau species are thought to adopt a corrupted, aggregate-prone conformation that seeds misfolding of native tau, leading to prion-like spread of tau aggregates throughout the brain (Holmes et al., 2014; Kaufman et al., 2016; Furman et al., 2017; DeVos et al., 2018). In addition, these tau “seeds” can propagate as distinct strains of misfolded protein, both within single patients and across different tauopathies (Mudher et al., 2017). In AD, amyloid-β (Aβ) species promote tau hyperphosphorylation and may also aid in spreading tau pathology by other mechanisms (Choi et al., 2014; Bennett et al., 2017; He et al., 2018).

There is strong clinicopathologic evidence that tau aggregation correlates with neuronal loss and cognitive decline both in AD (Arriagada et al., 1992; Nelson et al., 2012; Brier et al., 2016) and in tau transgenic animals (Santacruz et al., 2005; Wirths and Bayer, 2010; DeVos et al., 2017). However, whether tau aggregation is itself toxic or simply an epiphenomenon associated with toxic soluble tau species remains a matter of debate.

To visualize and measure proteopathic tau seeds, several different tau aggregation biosensor molecules have been developed. These reporters are composed of fluorescent molecules or other protein epitopes fused to the repeat domain of tau (TauRD), which has been altered to carry at least one proaggregation mutation such as MAPT P301L. Importantly, tau aggregation biosensors are designed to aggregate only in the presence of tau seeds, not spontaneously. The most established such model, composed of HEK cells expressing a TauRD biosensor, has been used to demonstrate the presence of tau seeds within donated human AD brain tissues as well as animal models of tauopathy (Holmes et al., 2014; Dujardin et al., 2020). Work with TauRD biosensors shows that seed-competent misfolded tau consists of high-molecular-weight phosphorylated oligomers and precedes neurofibrillary tangles in AD patient brain tissues (Holmes et al., 2014; Takeda et al., 2015, 2016; Kaufman et al., 2017; Nobuhara et al., 2017; DeVos et al., 2018; Dujardin et al., 2020).

The HEK TauRD reporter model has also been used to identify and propagate distinct morphologic strains of tau seeds from patients with AD and other tauopathies (Sanders et al., 2014; Kaufman et al., 2016, 2017). These strain morphologies have been described in HEK cells, but have not been observed in human neuronal cultures (Sanders et al., 2014). Interestingly, some morphologic strains of TauRD aggregates cannot be propagated in HEK cells because of apparent strain-specific cytotoxicity (Sanders et al., 2014).

In this study, we sought to create an accelerated human neuronal model of tau-induced neurotoxicity that incorporates both soluble tau species and seeded TauRD aggregation. We developed a model based on patient-derived induced pluripotent stem cell (iPSC) neurons that shows clear tau aggregate seeding and tau-induced toxicity after misfolded tau is taken up from the media. To accomplish this, we stably expressed a nonspontaneously aggregating TauRD biosensor in iPSC neurons and then seeded TauRD aggregation using tau seeds derived from mice carrying the MAPT P301L mutation (rTg4510; Ramsden et al., 2005; Santacruz et al., 2005). Using longitudinal *in vitro* live imaging and a custom single-cell tracking workflow, we were then able to compare neuronal survival between iPSC neurons that aggregated the TauRD construct and adjacent nonaggregating cells in the same well. This approach allows us to assess toxicity downstream of tau uptake and aggregation, controlling both for the expression of the TauRD construct and overall exposure to seed-competent tau.

The model also permits exploration of patient- and strain-specific neuronal responses to toxic tau species. Our recent studies have demonstrated that iPSC neurons carrying the familial AD (fAD)-causing PSEN1 L435F mutation show increased Aβ43, Aβ43/40, and Aβ42/40 ratios as well as increased phosphorylated tau compared with control iPSC neurons (Oakley et al., 2020). Based on prior reports showing that longer-length Aβ species promote TauRD seeding (Bennett et al., 2017), we hypothesized that that familial AD iPSCs would demonstrate accelerated seeding after the addition of exogenous tau species. Our observations support this notion. Following TauRD aggregation, there is an equivalent rate of subacute neuronal toxicity observed in both the PSEN1 mutant and control lines, suggesting that any effect of elevated Aβ43 in this model is on tau aggregation, rather than a direct effect on toxicity. Finally, we identify specific morphologic strains or patterns of TauRD reporter aggregates that appear to occur at different rates in the two cell lines and, interestingly, correlate with neuronal survival.

## Materials and Methods

### 

#### 

##### Plasmids and lentiviral production.

pLVX-TetOne-Puro-hNGN2 (human Neurogenin-2; Clontech) lentiviral plasmid for constitutive expression of puromycin resistance and doxycycline-inducible expression of human NGN2 was a gift from K.A. Worringer (Novartis Institutes for BioMedical Research, Boston, MA). Plenti-UBC-TauRD (P301L)-CFP-2A-TauRD(P301L)-YFP (yellow fluorescent protein) lentiviral plasmid was generated from pLenti-UBC (Thermo Fisher Scientific) using pcDNA3.1-TauRD(P301L)-CFP-2A-TauRD(P301L)-YFP (TauFRET2; Nicholls et al., 2017). Vesicular stomatitis virus glycoprotein (VSVG)-pseudotyped lentivirus was prepared and concentrated via ultracentrifugation as previously described (Dujardin et al., 2014).

##### Mouse brain lysate preparation.

Mouse brain lysates were prepared as previously described (Bennett et al., 2017). Whole brains from 12-month-old rTg4510 and control littermate mice (The Jackson Laboratory) were homogenized by dounce-homogenization in 5× weight by volume ice-cold PBS^−/−^ (Thermo Fisher Scientific) with protease inhibitor (catalog #5871S, Cell Signaling Technology) followed by centrifugation at 3000 × *g* for 10 min. The resulting pellets were resuspended in 500 µl PBS^−/−^ with protease inhibitor and sonicated on ice, then recentrifuged at 3000 × *g*. The resulting supernatant was used as seed material.

##### iPSC lentiviral transduction neuronal differentiation.

Lentiviral transduction was performed as previously reported with minor modifications (Oakley et al., 2020). To generate iPSCs expressing the TauRD reporter along with inducible NGN2 lentivirus, 300,000 iPSCs were plated on one well of a six-well plate in the presence of thiazovivin and coinfected with pLVX-TetOne-Puro-hNGN2 and Plenti-UBC-TauRD(P301L)-CFP-2A-TauRD(P301L)-YFP lentivirus overnight (O/N) at 24 h after plating. Following initial lentiviral transduction, cells were passaged with Accutase treatment into a 10 cm plate and selected with 5 µg/ml puromycin after 24 h (catalog #A11138-03, Thermo Fisher Scientific). Puromycin-resistant iPSCs were then expanded and kept under 5 µg/ml puromycin selection to limit lentiviral silencing.

Our initial approach to lentiviral infection was to use a TauRD-CFP_2A_TauRD-YFP split construct commonly used to produce a cyan fluorescent protein (CFP)-to-YFP FRET signal on tauRD aggregation (Holmes et al., 2014; Nicholls et al., 2017). However, after infecting iPSCs with lentivirus encoding ubiquitously expressed TauRD-CFP_2A_TauRD-YFP, we observed YFP expression only and a complete lack of CFP on flow cytometry (Extended Data Fig. 1-1*C*). Given the high level of sequence homology between the TauRD-CFP and TauRD-YFP halves of the construct, this result is most likely because of recombination during reverse transcription and has been observed in prior studies (Komatsubara et al., 2015). Furthermore, this was not the case in HEK293 cells infected with similar constructs, which showed both YFP-only and CFP/YFP double-positive cells (data not shown). Ultimately, our approach resulted in iPSCs expressing only the TauRD-YFP half of the construct, precluding the use of FRET to measure TauRD aggregation. Fortunately, the TauRD-YFP signal alone can be used to monitor TauRD aggregation via standard epifluorescent or confocal microscopy.

iPSC neurons were subsequently differentiated using doxycycline-driven expression of hNGN2 combined with SMAD and WNT inhibition, as previously described, with the continued use of antimitotic for the duration of experiments to reduce dividing non-neuronal cells during live imaging (Nehme et al., 2018; Oakley et al., 2020). At day 0 (D0), NGN2-inducible iPSCs were passaged as single cells with Accutase and plated at 400,000 cells/well on Matrigel-coated six-well plates in mTeSR medium with rock inhibitor (1 μm; thiazovivin, EMD Millipore). Doxycycline (2 μg/ml; Sigma-Aldrich) was added on plating at D0 to induce NGN2 expression and maintained in culture medium thereafter. On D1, medium was switched to N2 media [DMEM:F12 (Thermo Fisher Scientific), Glutamax (1%; Thermo Fisher Scientific), dextrose (0.3%; EM Science), N2 (1%; Thermo Fisher Scientific)] supplemented with SB431542 (10 μm; Tocris Bioscience), LDN-193189 (100 nm; Stemgent), XAV939 (2 μm; Stemgent), and doxycycline. On D2, cells were fed with N2 medium with SB/XAV/LDN at one-half the concentration on D1 and doxycycline at full concentration. On D3, cells were fed with N2 medium supplemented with NT3 (10 ng/ml; PreproTech), BDNF (10 ng/ml; R&D Systems), GDNF (10 ng/ml; R&D Systems), and doxycycline. On D4, cells were switched to NBM media [Neurobasal Medium, minus phenol red (Thermo Fisher Scientific), Glutamax (1%), dextrose (0.3%), NEAA (0.5%; Thermo Fisher Scientific), and B27 (2%; Thermo Fisher Scientific)] supplemented with NT3, BDNF, GDNF, doxycycline, and the antimitotic FUDR (floxuridine; 10 μm; Sigma-Aldrich). Cells were fed with D4 media every 2–3 d until day 14, and cells were fed weekly thereafter. Beginning at D8, 2% horse serum (catalog #26–050-088, Thermo Fisher Scientific) was added to media to improve neuronal survival. For live imaging and immunohistochemistry, differentiating neurons were passaged at D7 using Accutase treatment and plated onto 96-well plates (20,000 cells/well) with 2% horse serum added at this time to improve survival. The 96-well plates were precoated with poly-d-lysine (catalog #356640, Corning) and further coated with laminin (10 µg/ml; Sigma-Aldrich), fibronectin (2 µg/ml; Sigma-Aldrich), and Matrigel (2.5×; Corning) in DMEM:F12 for 3 h at room temperature (RT).

##### Species and sex studied.

The human iPSC lines used in these studies are all female (Table 1).

**Table 1.** Clinical characteristics of iPSC donors

Case	Age (years)	Sex	Diagnosis	Mutation	Braak stage	Thal phase	CERAD density	Diffuse Aβ	ApoE	Race
2012, C1	>90	F	Control	N/A	II	0	0	None	3/3	W
2048, fAD1	53	F	Familial AD	PSEN1 L435F (c.1303C>T)	VI	3	3	Frequent	3/3	W

Braak, Thal, and CERAD staging as described (Hyman et al., 2012). AopE, ApoE genotype; F, female; W, white; CERAD, Consortium to Establish a Registry of Alzheimer's Disease.

##### Live imaging experiments.

iPSC neurons expressing TauRD-YFP reporter were cultured as described above and plated at day 7 of differentiation onto 96-well plates, where they were allowed to mature until day 14–17 of differentiation before live-imaging experiments. Four hours before imaging, a far-red nuclear dye (NucSpot Live 650, catalog #40082, Biotium) was added to the media to allow time-lapse visualization of nuclear morphology and nuclear fragmentation associated with cell death (1:1000 in DMSO). At this time, DNAse1 (catalog #LK003170, Worthington Biochemical Corporation) was also added at a final concentration of 0.148 U/µl to predigest dead cell nuclei, which can otherwise obscure the signal from live nuclei. Within one imaging interval before the start of imaging (2 h), tau seed-containing and control brain lysates were added to the culture media at 1–10 µg of total protein into 150 µl media/well. Epifluorescent live imaging was then begun using a temperature and CO_2_ controlled microscope (Cytation5, BioTek) with images taken every 2 h for 7 d. For each well imaged, a 4 × 4 or 5 × 5 grid of 10× images was acquired. Following live-imaging experiments, media was harvested, and cells were fixed for immunohistochemistry.

##### Flow cytometry.

Flow cytometry to measure expression of TauRD reporter was performed using a MACSQuant VYB Flow Cytometer (Miltenyi Biotec) equipped with VivoBlue (CFP) and FITC (YFP) imaging channels.

##### iPSC/iPSC neuron immunocytochemistry and microscopy.

Immunocytochemistry was performed as previously described (Oakley et al., 2020). Stained cells were imaged on a BioTek Cytation 5 or ImageXpress Micro Confocal Microscope. YFP staining was performed using a cross-reacting chicken anti-GFP antibody (1:500; catalog #ab13970, Abcam), PHF1 anti-phospho-tau antibody (1:200) was a gift from the Davies Laboratory. Immunocytochemistry for Tuj1 and cortical layer markers was performed using antibodies against Tuj1 (1:500; TUJ, Aves), CUX1/CUTL1 (1:300; catalog #ab54583, Abcam), Brn2 (1:300; catalog #ab1377469, Abcam), Ctip2 (1:100; catalog #ab18465, Abcam), and Tbr1 (1:1000; catalog #ab31940, Abcam).

##### Image analysis and neuronal survival.

Following live-imaging experiments, resulting images were processed, and neuronal survival scored using a toolset of custom NIH ImageJ macros. The macro toolset facilitates image preprocessing (background subtraction, time-lapse image alignment, fluorescent channel overlay), manual cell scoring of morphologically identified neurons (frame-by-frame assignment of cell survival, cell position, and aggregate formation), censoring of cells that leave the field of view, and recording results. Measurement of TauRD reporter seed formation in neurites (see Fig. 3) was performed using Cytation Gen3 software to identify objects 0.1–4 µm in size >10,000 gray levels above local background. Larger bright areas were excluded from the analysis to avoid overcounting within clusters of cells (Extended Data Fig. 2-1)

Automated WEKA segmentation was used to measure neurite density of iPSC neurons. Trainable WEKA segmentation (version 23.2.32) with a fast random forest classifier was used to classify GFP images into the following three compartments: cell bodies, neurites, and background. The following training features used were: Gaussian blur, Hessian, membrane projections, Sobel filter, and difference of Gaussians with settings membrane thickness = 1, membrane patch size = 19, and σ = 1–16. A probability map of the neurite compartment was thresholded using NIH ImageJ to produce object-level information for neurite area measurements.

The percentage of Tuj1 positivity following live-imaging experiments was determined using Gen5 software (BioTek) following immunohistochemistry as described above. Cortical layer marker expression in neurons was analyzed using custom NIH ImageJ macros. Neuronal nuclei were first identified and annotated based on Tuj1 positivity and DAPI staining in a manner analogous to neuronal identification in live-imaging experiments. This was done to ensure similarity to the populations analyzed in neuronal seeding and survival assays. Subsequently, individual neuronal nuclei were segmented from images, DAPI stain was used to mask nuclear compartments, and underlying RFP and CY5 positivity was measured using thresholding and particle analysis in NIH ImageJ. Results were then processed and visualized using Excel and R Studio.

Strain morphologies in seeded iPSC neurons were identified in a blinded fashion using morphologic categories as described previously (Sanders et al., 2014). The 70 × 70 pixel images of tracked neurons with aggregates (YFP channel only) were extracted and displayed as animated *z*-stacks, restricted to frames starting at the time of aggregate formation to either the end of the experiment or cell death, whichever occurred sooner. Images were chosen randomly from all tracked neurons in four biological replicates. Cell line or neuronal life span was not viewable at the time of scoring. Choices of strains were “Ordered,” “Disordered,” “Speckles,” “Toxic,” “Other,” or “Unclassifiable.” Unclassifiable cells were subsequently excluded from analysis. The Speckles category was interpreted to mean prominent nuclear speckles as opposed to diffuse cytoplasmic speckling, which fell into the category of Disordered. Subsequent data analysis was performed in R Studio.

##### Brain lysate labeling and uptake assay.

Lysate was labeled with Alexa Fluor 647-NHS (catalog #A37573, Thermo Fisher Scientific) for 1 h at RT, according to the manufacturer instructions. The resulting mixture was then dialyzed in PBS O/N at 4°C using a 2 kDa cutoff membrane (catalog #66205, Thermo Fisher Scientific) to remove unbound label. Labeled lysate was added to iPSC neurons at 10 µg of total protein/well and subsequently incubated for 5 d, before fixation with 4% paraformaldehyde. Fixed neurons were labeled with DAPI and imaged on an ImageXpress Micro Confocal Microscope in DAPI, FITC (YFP), and CY5 (Alexa Fluor 647) channels at 20× with 20 *z*-planes. The resulting maximal intensity projection whole-well tiled images were then analyzed using custom ImageJ macros. Neurons were identified and outlined in the YFP channel and assigned to either aggregate-bearing or non-aggregate-bearing classes. The mean Alexa Fluor 647 intensity in the neuronal soma and DAPI-labeled nuclear compartment was subsequently measured manually for each neuron. Results were analyzed in R studio using the ggplot2 library.

##### ELISA.

ELISA measurements of Aβ species were run according to the manufacturer instructions as previously described (Oakley et al., 2020). Human Aβ 1–40 (catalog #298–64601, WAKO), human Aβ 1–42 (catalog #296–64401, WAKO) and human Aβ 1–43 (catalog #27710, IBL) were measured separately in centrifuged cell supernatant. Assay plates were analyzed using a PerkinElmer Wallac Victor2 at 450 nm.

##### Western blot.

Cells were harvested at D28 on ice into ice-cold RIPA buffer with protease inhibitor (catalog #5871S, Cell Signaling Technology) using a cell scraper. Subsequently, cells were lysed with 10 passes using insulin syringes and pelleted for 10 min × 10 kg at 4°C. Western blots were performed on 10 μg samples of soluble supernatants using the Invitrogen NuPage Novex Gel System (Thermo Fisher Scientific) according to the manufacturer instructions. Fluorescent secondary antibodies were used at a concentration of 1:5000. Blots were imaged on a LI-COR Odyssey system and analyzed using LI-COR Image Studio.

##### Statistical methods.

Calculation of SD, SE, and two-tailed Student's and Welch two-sample *t* tests were performed using Microsoft Excel Analysis ToolPak and R. Kaplan–Meier survival curves, log-rank tests, and Cox proportional hazards were calculated using the R survminer package. Two-way ANOVA and *R*^2^ values were calculated using the R rstatix package.

## Results

### TauRD reporter expression in control and fAD human iPSC-derived neurons

Two iPSC lines derived from brain donors at the time of autopsy were selected for use in this study: one from a >90-year-old cognitively normal female (MADRC_2012, C1) and one from a 53-year-old female fAD patient carrying the PSEN1 L435F mutation (MADRC_2048, fAD1; Table 1; Oakley et al., 2020). Both patients underwent complete neuropathologic evaluation to confirm diagnosis, and both were apolipoprotein E (ApoE) 3/3 genotype (Table 1; Hyman et al., 2012).

**Figure 1.**
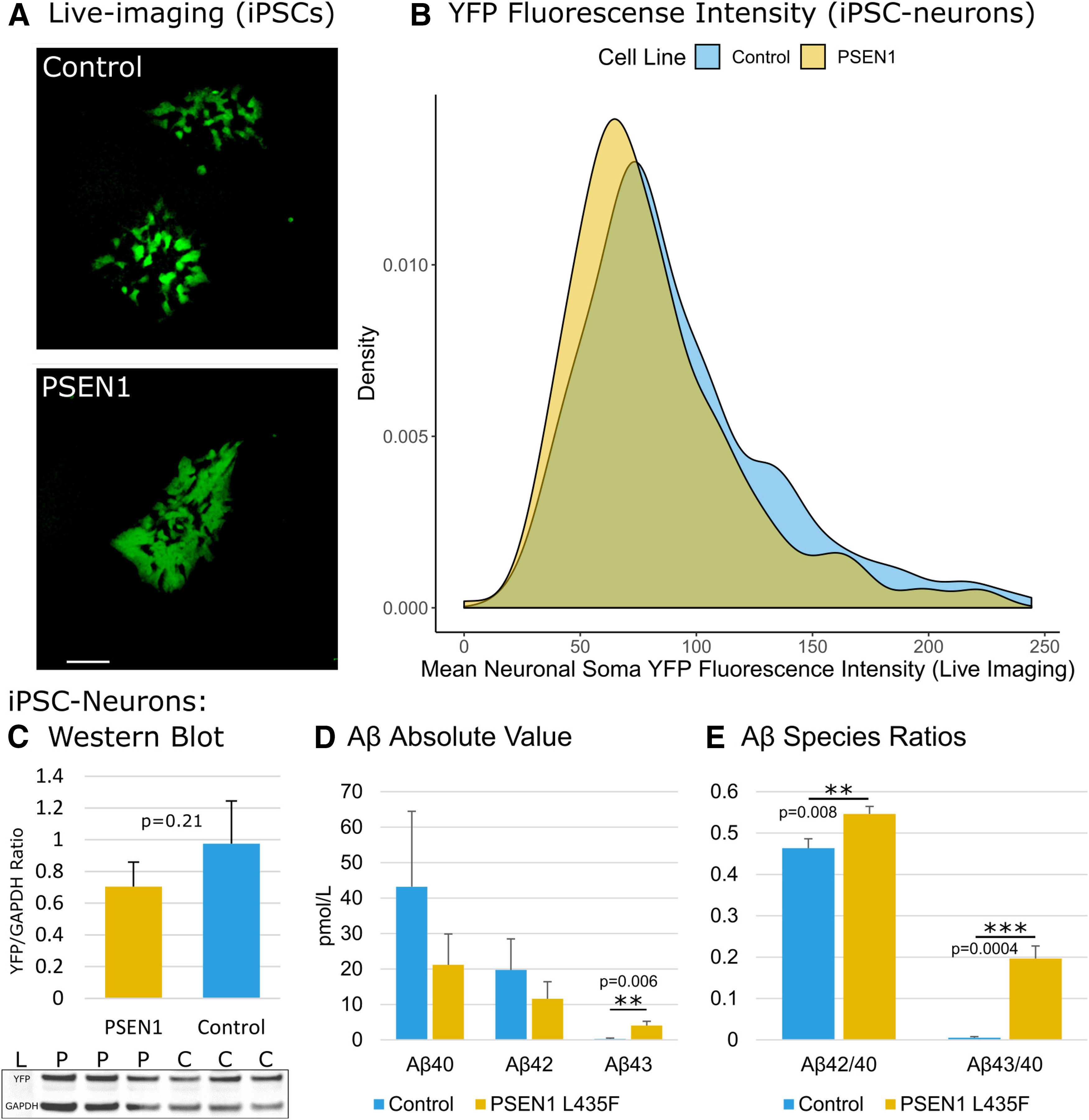
TauRD-YFP reporter and Aβ expression. ***A***, Unstained fluorescent image of control and PSEN1 iPSC colonies showing partial expression of TauRD reporter. Scale bar, 100 µm. ***B***, Density distribution of neuronal soma YFP fluorescence intensity measured by live imaging of iPSC neurons at the end of differentiation (day 15): control = 255 YFP-positive neurons; PSEN1 = 228 YFP-positive neurons. This analysis includes all YFP-expressing neurons visible on epifluorescent imaging. ***C***, Western blot results demonstrating equivalent expression of the TauRD-YFP construct in PSEN1 L435F and control iPSC-derived neuronal cultures (*n* = 3 independent neuronal differentiations, day 28, mean ± SD). ***D***, ***E***, Secreted Aβ43 species and Aβ42/Aβ40 and Aβ43/Aβ40 ratios are elevated in PSEN1 L435F iPSC neurons (mean ± SD). ***D***, Absolute Aβ levels in media for control and PSEN1 mutant iPSC neurons (D28 media, ELISA, ± SD). ***E***, Aβ42/Aβ40 and Aβ43/Aβ40 ratios (±SD; Extended Data Figures 1-1, 1-2).

Neuronal differentiation of iPSCs was performed using stable lentiviral transduction with a puromycin-selectable NGN2 construct combined with SMAD and WNT inhibition (Nehme et al., 2018; Oakley et al., 2020). We took advantage of this viral approach and coinfected control and PSEN1 iPSCs with both the inducible NGN2 lentivirus and a second TauRD reporter lentivirus at the time of stable line generation. We reasoned that epigenetic events preventing silencing of the puromycin-selectable NGN2 lentivirus might also limit silencing of the coinfected TauRD reporter. This was indeed the case. Both resulting polyclonal NGN2/TauRD-YFP reporter iPSC lines expressed the TauRD-YFP reporter in a subset of cells for over 10 passages under puromycin selection without apparent decrease in expression level (Fig. 1*A*, Extended Data Fig. 1-1*C*; see Materials and Methods). Both iPSC lines generated in this fashion also maintained a normal karyotype (Extended Data Fig. 1-1*A*,*B*). When measured by flow cytometry, each iPSC line expressed equivalent levels of the TauRD-YFP reporter in a similar percentage of iPSCs (Extended Data Fig. 1-1*C*). After differentiation, YFP-expressing iPSC neurons showed equivalent levels of reporter fluorescence between the two lines (Fig. 1*B*; see below). Furthermore, neuronal cultures derived from the control and PSEN1 TauRD reporter lines express equivalent levels of the TauRD-YFP reporter compared with GAPDH on Western blot (Fig. 1*C*). The percentage of neuronal cells produced as measured by Tuj1 positivity was not significantly different between control and PSEN1 mutant lines (Extended Data Fig. 1-2*A*,*D*). Prior studies show that the neurons produced by NGN2 induction in iPSCs are excitatory layer II/III cortical neurons (Zhang et al., 2013; Nehme et al., 2018). We confirmed this finding in our iPSC neurons using immunohistochemistry for the upper-layer cortical markers Cux1 and Brn2, lower-layer cortical marker Ctip2 (negative staining), and the glutamatergic neuron marker Tbr1 (Extended Data Fig. 1-2*B*,*C*,*E*).

10.1523/JNEUROSCI.2590-20.2021.f1-1 Figure 1-1 Karyotype and Flow Cytometry on iPSCs. ***A***, ***B***, Normal karyotype of control (***A***) and PSEN1 L435F (***B***) iPSCs expressing the TauRD reporter. ***C***, Flow cytometry of control (top) and PSEN1 (bottom) iPSCs demonstrating similar overall levels of TauRD-YFP expression and an absence of CFP-TauRD expression (attributed to recombination during reverse transcription and viral integration). Download Figure 1-1, EPS file.

**Figure 2.**
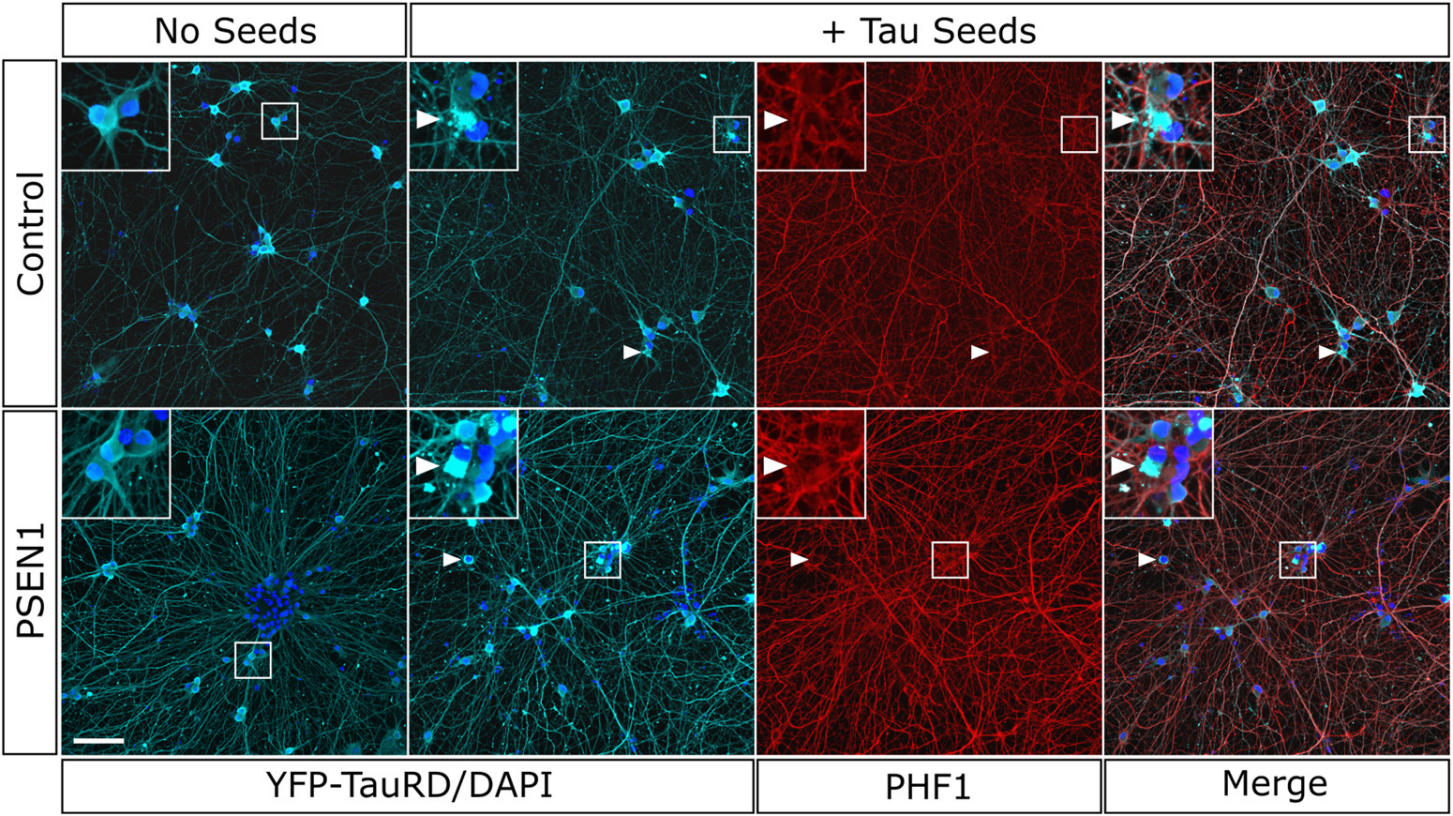
TauRD-YFP reporter expression in iPSC neurons and aggregation in response to misfolded tau seeds. Control and PSEN1 mutant iPSC neurons as indicated with and without exposure to tau seeds for 7 d before fixation and immunohistochemistry for DAPI (blue), YFP (cyan), and phospho-tau (PHF1, red). Following exposure to tau seeds, iPSC neurons develop aggregates of TauRD-YFP reporter (arrowheads), shown at higher magnification in inset. Scale bar, 50 μm (Extended Data Figure 2-1).

10.1523/JNEUROSCI.2590-20.2021.f1-2 Figure 1-2 NGN2-directed differentiation produces high-purity layer II/III glutamatergic cortical neurons. ***A***, Neurons immunostained for the neuronal marker Tuj1 following live-imaging experiments (*n* = 4 replicates matched to those used for live-imaging experiments, without addition of brain lysate). Nuclear staining is Nucspot650 as used in live imaging. ***B***, ***C***, Neurons immnostained at day 14 of differentiation for upper-layer cortical markers Cux1 and Brn2 (***B***), lower-layer cortical marker Ctip2 (***C***), and the glutamatergic neuron marker Tbr1 (***C***). Day 14 chosen to represent the population of neurons present at the start of live imaging experiments. Scale bars: ***A***, 200 µm; ***B***, ***C***, 100 µm. ***D***, Quantification of TUJ1-positive cells as a percentage of all NucSpot 650-stained nuclei at day 21. The *p* values are for control versus PSEN1 comparison (*n* = 4 independent differentiations). ***E***, Quantification of neuronal fate markers in day 14 TUJ1-positive neuronal nuclei shows a high percentage of BRN2-, CUX1-, and TBR1-positive neurons consistent with layer II/III glutamatergic cortical neurons. The *p* values are for control versus PSEN1 comparison (*n* = 2 independent differentiations). Error bars indicate ±SE. Download Figure 1-2, EPS file.

Following the generation of NGN2/TauRD-YFP iPSCs from control and fAD lines, iPSC neurons were produced and secreted levels of Aβ were measured. Consistent with prior results, neurons derived from the fAD PSEN1 L435F iPSC line produced significantly higher levels of Aβ43 species, and had higher Aβ43/40 and Aβ42/40 ratios, compared with control iPSC neurons (Fig. 1*D*,*E*; Oakley et al., 2020).

### Tau seeding in control and fAD human iPSC-derived neurons

**Figure 3.**
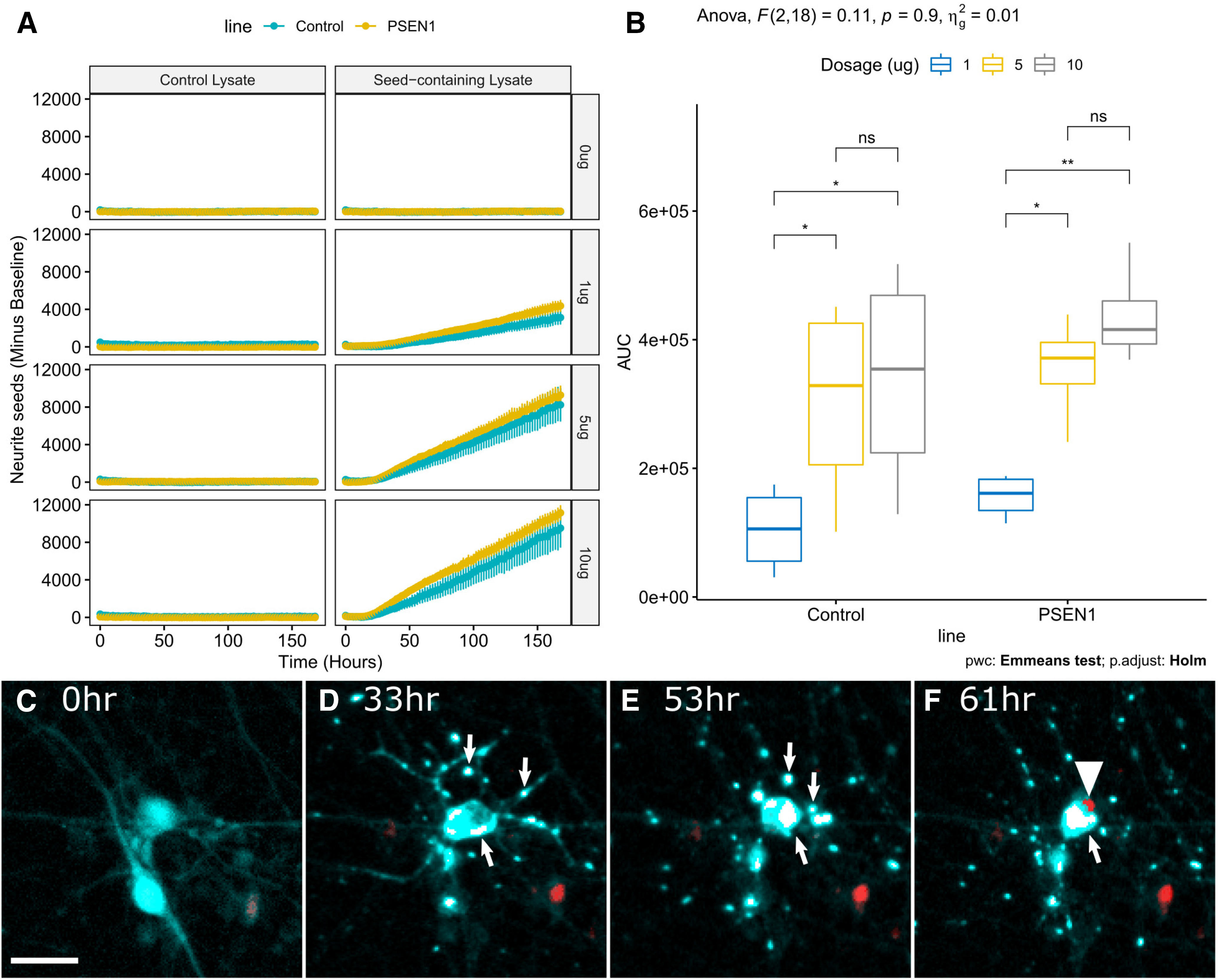
Live imaging of neurite seeding and cell death in iPSC neurons. ***A***, Graphs depict the average number of TauRD-YFP reporter seeds within neurites per well for 7 d following the addition of either seed-containing lysate (rTg4510) or control lysate (±SE). *n* = 3 independent neuronal differentiations for all experiments except control neurons paired with 1ug control lysate where *n* = 2. Values represent the sum of two technical replicates per experiment. Subtracted baseline is the overall average number of events detected in the no-lysate condition for each line (quantification noise). ***B***, Mean area under the curve for neurite seeding assay in ***A*** plotted for each dosage of seed-containing lysate, grouped by cell line. *p ≤ 0.05, **p ≤ 0.01. Two-way ANOVA results at top are for the interaction of cell line and dosage (n.s., *p* = 0.9). ***C–F***, Following the addition of tau seeds at 0 h (***C***), TauRD-YFP aggregation occurs beginning in neurites (arrows; ***D***), concentrating in cell body (***E***), and preceding neuronal death, highlighted by increased red signal from nuclear dye (arrowhead; ***F***). The cell body disappears following cell death. Cyan, TauRD-YFP; white, Tau-YFP aggregates (saturated, pseudocolored); red, NucSpot 650 showing maximal signal at cell death. PSEN1 mutant iPSC neurons used in this example. Scale bar, 25 μm (Extended Data Figures. 3-1, 3-2).

TauRD aggregation was induced in both control and PSEN1 L435F mutant iPSC neurons by adding phosphorylated tau seeds to the culture media. iPSC neurons derived from both lines were exposed to brain lysate from mice overexpressing P301L mutant tau protein (rTg4510) and control littermates. The rTg4510 mice serve as a uniform, highly concentrated source of phosphorylated tau seeds that are taken up into neurons rapidly (within 24 h) and induce aggregation of TauRD reporters (Takeda et al., 2015). Following treatment of iPSC neurons with tau seeds in the absence of lipofectamine, TauRD reporter aggregates were observed to form within neurites and cell bodies in both cell lines within ∼15 h (Figs. 2, 3*A*). No spontaneous TauRD reporter aggregation was noted in neurons from either iPSC line (Fig. 2) and has not been observed in cultures up to 6 months of age (data not shown). Control mouse brain lysate did not induce TauRD reporter aggregation (Fig. 3*A*). Prior work has indicated that there may be templated misfolding and coaggregation of endogenous tau species along with TauRD constructs (Reilly et al., 2017). To assess for this possibility, immunocytochemistry for endogenous phosphorylated tau was performed using a phospho-tau-specific antibody that binds outside the TauRD domain present in the reporter (PHF1). PHF1 antibody demonstrated extremely weak, if any, PHF1 incorporation within TauRD aggregates at 7 d after seed exposure (Fig. 2, inset).

10.1523/JNEUROSCI.2590-20.2021.f2-1 Figure 2-1 Alexa Fluor 647-labeled rTg4510 brain lysate uptake and seeding at 5 d post-seed addition in control iPSC neurons. ***A***, TauRD aggregate formation (green) in response to labeled rTg4510 brain lysate addition into culture media (red). Nuclei are labeled with DAPI (Blue). ***B***, Labeled rTg4510 lysate only from ***A***. ***C***, Control condition identical to ***A*** except without the addition of labeled rTg4510 seed material. ***D***, Red channel only from ***C*** (no rTg4510 lysate). ***E–G***, Alexa Fluor 647-labeled rTg4510 lysate uptake measurement in TauRD reporter-expressing iPSC neurons. ***E–G***, Average Alexa Fluor 647-labeled intensity was calculated for the entire neuronal soma (***E***), the soma minus the nuclear compartment (***F***), and the nuclear compartment alone (***G***). Comparison of average intensity was then performed for TauRD aggregate-bearing neurons (*n* = 26, Aggregate) versus nonaggregate-bearing neurons (*n* = 65, NoAgg). No significant differences in Alexa Fluor 647-labeled uptake were identified between the two classes. Scale bar, 20 μm. Download Figure 2-1, EPS file.

10.1523/JNEUROSCI.2590-20.2021.f3-1 Figure 3-1 Quantification of neurite aggregates: example 10× fields of TauRD reporter-expressing iPSC neurons immediately after (time 0) and 130 h after (time 65) addition of rTg4510 lysate. Cyan regions of interest indicate detected neurite aggregates. Objects at time 0 or without addition of lysate are false positives and are subsequently subtracted from counts. Large objects (cell bodies, cell clusters, single large aggregates) are excluded from the analysis. Scale bar, 200 µm. Download Figure 3-1, EPS file.

10.1523/JNEUROSCI.2590-20.2021.f3-2 Figure 3-2 Neurite density in control and PSEN1 neurons. Top, Original and WEKA segmented images as indicated. Gray values in the lower row represent the probability of segmentation into the background or neurite categories. Segmentation in the top right image is created by thresholding the neurite probability without local background subtraction. Bottom, Quantification of average neurite area per image in neurite seeding assay ± SD (*n* = 3 independent neuronal differentiations, samples are matched to those in the neurite seeding assay and those with supernatant collected for Aβ species measurement). Download Figure 3-2, EPS file.

TauRD aggregates formed only in a subset of neurons following exposure to seed material. To assess whether or not this cell-by-cell difference was reflective of variable seed uptake, fluorescently labeled rTg4510 lysate was applied to iPSC neurons. Following a 5 d incubation to allow seeded TauRD aggregation, the neuronal uptake of the fluorescent label (Alexa Fluor 647) was measured in the cytoplasmic and nuclear compartment of neurons with and without TauRD aggregates. We detected no difference in Alexa Fluor 647-labeled uptake between neurons with and without TauRD aggregates in either the cytoplasmic or nuclear compartments (*p* = 0.3 for the neuronal soma as a whole; *p* = 0.19 for the cytoplasmic compartment; and *p* = 0.89 for the nuclear compartment; Extended Data Fig. 2-1). While these results suggest that there is no difference in brain lysate exposure between TauRD aggregating and nonaggregating cells, it is possible that more subtle, tau-specific uptake varies among cells.

To understand the time course of induced TauRD aggregation and its potential influence on neuronal survival, an *in vitro* live-imaging assay was developed to monitor TauRD aggregate formation. Following the addition of tau seeds, epifluorescent live imaging was begun within 2 h using a temperature- and CO_2_-controlled microscope (Cytation5, BioTek) with images taken every 2 h for 7 d. A far-red nuclear dye (NucSpot 650, Biotium) was included in the media to allow time-lapse visualization of nuclear morphology and nuclear fragmentation associated with cell death. In iPSC neurons, NucSpot 650 lightly labels the nuclei of living neurons and brightly labels nuclei following cell lysis. Beginning ∼15 h after seeding with rTg4510 lysate, we observe the formation of TauRD aggregates in the soma and neurites of iPSC neurons (Fig. 3). These aggregates indicate neuronal uptake of misfolded tau and subsequent seeded reporter aggregation.

Live imaging was performed on paired cultures of control and PSEN1 TauRD-YFP expressing iPSC neurons following the addition of varying concentrations of control and rTg4510 brain lysates. TauRD-YFP aggregates within neurites tended to be smaller than those that accumulate within cell bodies (Figs. 2, 3*C–F*). We took advantage of this difference to develop an automated quantification of TauRD-YFP aggregates within neurites (objects <4 µm in size; Extended Data Fig. 3-1). We then applied the automated quantification to control and PSEN1 L435F neuronal cultures treated with control and seed-containing brain lysates over a range of concentrations (Fig. 3*A*). Tau seed-containing brain lysate induced increasing numbers of TauRD-YFP aggregates over the course of 7 d when applied at concentrations of 1, 5, or 10 µg of total protein/well in a 96-well plate format (150 µl media/well; Fig. 3*A*). Control brain lysates failed to induce TauRD-YFP aggregation under the same conditions (Fig. 3*A*). Two-way ANOVA was then performed to evaluate the effect of cell line identity and rTg4510 brain lysate dosage on the area under the curve (AUC) depicted in Figure 3*A* (results of 1, 5, and 10 µg rTg4510 conditions). There was a significant effect of rTg4510 lysate dosage on AUC (*F*_(2,18)_ = 11.3, *p* = 0.0007), but not of cell line identity (*F*_(2,18)_ = 2.2, *p* = 0.159), and there was no significant interaction between cell line identity and lysate dosage (*F*_(2,18)_ = 0.11, *p* = 0.90; Fig. 3*B*). Pairwise comparisons of AUC as a function of lysate dosage for each line are depicted in Figure 3*B*. The percentages of Tuj1-positive cells and average neurite density were not significantly different between the control and PSEN1 iPSC neuron cultures at the end of live-imaging assays (Extended Data Figs. 1-2*A*,*D*, 3-2).

Following formation, aggregates are trafficked within neurites and growth cones, and accumulate in cell bodies (Fig. 3*C–F*, Movie 1). We observed that some aggregate-containing cells underwent nuclear fragmentation and cell death, highlighted by a large increase in fluorescence intensity of the far-red nuclear dye, and thus were also able to monitor the temporal relationship of aggregate formation and cell death in individual neurons (Fig. 3*C–F*, Movies 1, 2, 3).

**Figure 4.**
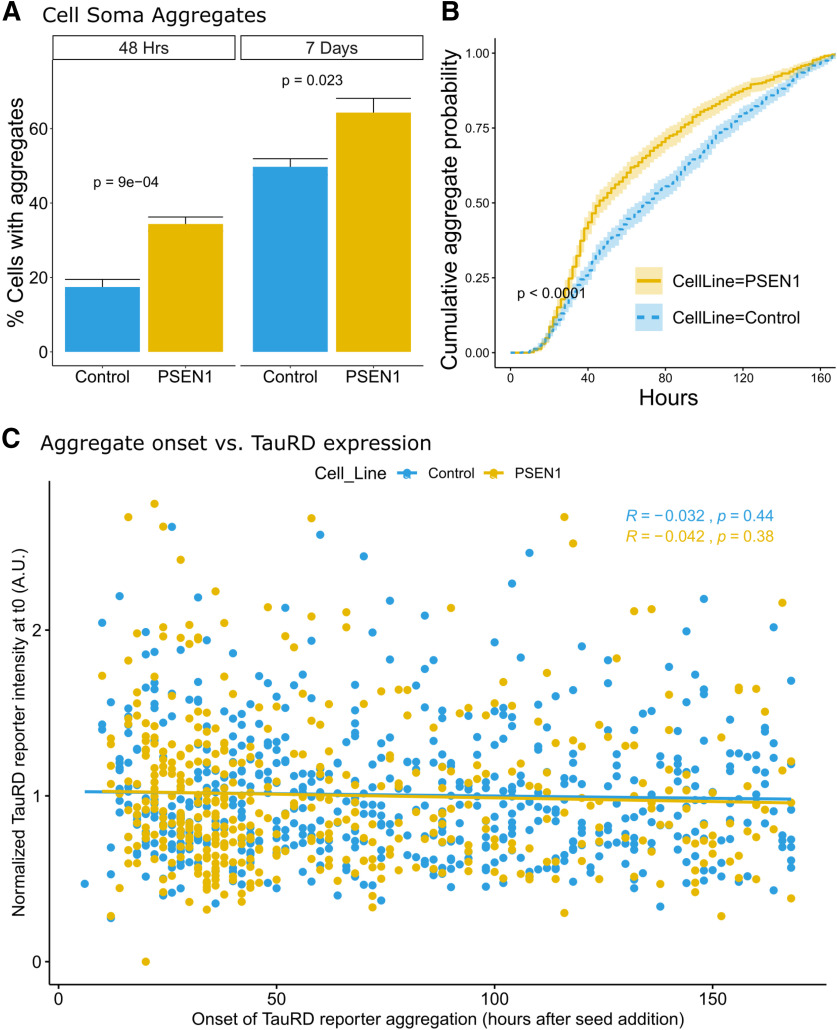
Accelerated aggregate formation in PSEN1 mutant iPSC neurons. ***A***, PSEN1 L435F mutant neurons show a higher percentage of TauRD-YFP cell soma aggregates compared with control at both 48 h and 7 d after addition of 10 µg seed material (*p* = 0.0009 and *p* = 0.0232 respectively; *n* = 4 independent neuronal differentiations, ±SE). ***B***, Time course of soma aggregate formation in control and PSEN1 L435F mutant iPSC neurons (*p* < 0.0001, plots are ±95% confidence interval). ***C***, Onset of TauRD reporter aggregation does not correlate with expression levels. The onset of TauRD reporter aggregation is plotted against normalized fluorescence intensity of the TauRD reporter per cell at the first imaging time point (t0). Pearson correlation is plotted for each cell line. *n* = 3 independent differentiations. In total, 591 control neurons and 441 PSEN1 mutant neurons are depicted. Normalized t0 values correspond to the average TauRD fluorescence intensity in a region of interest centered over the tracked neuron divided by the average fluorescence intensity of all tracked neurons in each replicate per cell line. Neurons that do not develop TauRD aggregates are excluded from this analysis (Extended Data Figure 4-1).

Movie 1. Video of aggregate formation and neuronal death in PSEN1 mutant neuron from Figure 4. Imaging begins within one imaging interval (2 h) following the addition of 10 μg of rTG4510 brain lysate. Cyan, TauRD reporter; red, NucSpot 650. Flash of increased NucSpot 650 brightness indicates membrane lysis and neuronal death. 10.1523/JNEUROSCI.2590-20.2021.video.1

10.1523/JNEUROSCI.2590-20.2021.f4-1 Figure 4-1 Scored live imaging sample. Individual neuronal cells manually tracked using a custom ImageJ macro throughout the duration of a 7 d live-imaging experiment and scored according to their ultimate phenotype. Image above is the first time point of live imaging. Cell class names in symbol key are composed of Initial_State+End_State+Survival_Status (i.e., O + A + D above) and colored yellow if censored during the live imaging experiment. For Initial_State and End_State A=TauRD reporter aggregate, O is no aggregate. For Survival_Status, A is Alive and D is Dead. “Trans” indicates transient aggregation. Cyan, YFP-TauRD reporter; red, NucSpot 650 Nuclear dye. Download Figure 4-1, EPS file.

### fAD iPSC neurons show accelerated tau seed formation

Because fAD-causing mutations in PSEN1 are thought to accelerate the formation of age-related neurofibrillary tangles within neurons, we next sought to determine whether or not PSEN1 L435F neurons exhibited differences in the profile of TauRD-YFP seed formation in the live-imaging assay (Oddo et al., 2003; Sperling et al., 2014). Measurement of seed formation in neurites indicates that there is a nonsignificant trend toward more TauRD neurite seeds in the PSEN1 L435F cell line (Fig. 3). In cultures treated with the highest dose of seed material, there was an apparent early acceleration of seed formation in neurites 24–48 h after seed addition, with a significantly higher slope of the seeding curve between these time points in PSEN1 mutant cells compared with controls (*p* = 0.016). Differences in the slope of seeding were not seen in conditions with lower concentrations of seed material, and they were not present at later time points in the assay.

To extend this analysis, single-neuron tracking macros were developed for NIH ImageJ to facilitate manual longitudinal phenotyping of individual cells throughout the course of live-imaging experiments (Extended Data Fig. 4-1). Using this software, all individually identifiable YFP-positive cells with neuronal morphology in the high-dose seed condition were tracked, and the presence of TauRD-YFP aggregates in the cell soma was evaluated per time point (*n* = 1598 control neurons and *n* = 983 PSEN1 neurons from four independent neuronal differentiations). Cell death was highlighted using a far-red nuclear dye (NucSpot 650) and recorded when present (Movies 2, 3). Neurons that left the field of view during the course of the experiment were classified as censored at that time point for the purpose of subsequent data analysis.

Movie 2. Video of the first 100 cell deaths in control iPSC neurons, with or without TauRD aggregation. Imaging begins within one imaging interval (2 h) following addition of 10 μg of rTG4510 brain lysate. Cyan, TauRD reporter; red, NucSpot 650. Flash of increased NucSpot 650 brightness indicates membrane lysis and neuronal death. Data are combined from four independent neuronal differentiations. 10.1523/JNEUROSCI.2590-20.2021.video.2

Movie 3. Video of the first 100 cell deaths in PSEN1 L435F iPSC neurons, or and without TauRD aggregation. Imaging begins within one imaging interval (2 h) following addition of 10 μg of rTG4510 brain lysate. Cyan, TauRD reporter; red, NucSpot 650. Flash of increased NucSpot 650 brightness indicates membrane lysis and neuronal death. Data are combined from four independent neuronal differentiations. 10.1523/JNEUROSCI.2590-20.2021.video.3

Single-cell tracking revealed accelerated cell soma TauRD-YFP aggregate formation in PSEN1 mutant iPSC neurons treated with tau seeds compared with control. Forty-eight hours after seed material addition, a greater percentage of PSEN1 mutant neurons contained cell soma aggregates compared with controls [34.4 ± 3.8% (mean ± SD) in PSEN1 vs 17.4 ± 4.1% in control neurons *p* = 0.0009; Fig. 4*A*]. A higher percentage of soma aggregate-containing neurons was also observed in PSEN1 mutant neurons at the end point of the 7 d experiment (64.3 ± 7.7% in PSEN1 vs 49.7 ± 4.3% in control neurons; *p* = 0.023; Fig. 4*A*). The overall number of neurons counted was not significantly different between the two lines (*p* = 0.129). Furthermore, cumulative event analysis showed that cell soma aggregation tended to occur earlier in PSEN1 mutant neurons compared with control (*p* < 0.001; Fig. 4*B*).

Because it is possible that subtle variation in the expression level of the TauRD reporter might underlie a portion of the observed differences between control and PSEN1 neurons in this assay, we then measured the average reporter fluorescence intensity in neuronal somas at the beginning of the assay [time 0 (t0)] and correlated this with the subsequent onset of TauRD reporter aggregation on a per cell basis (Fig. 4*C*). There was no significant difference in the average t0 neuronal soma fluorescence intensity between the two cell lines (average PSEN1/control ratio = 0.88 ± 0.08, *p* = 0.082, *n* = 3). Additionally, there was no relationship between TauRD reporter expression and the onset of reporter aggregation in either the control or PSEN1 cell lines (control: *R*^2^ = −0.032, *p* = 0.44; PSEN1: *R*^2^ = −0.042, *p* = 0.38; Fig. 4*C*).

### Neuronal cell death follows tau seed formation in both control and fAD iPSC neurons

**Figure 5.**
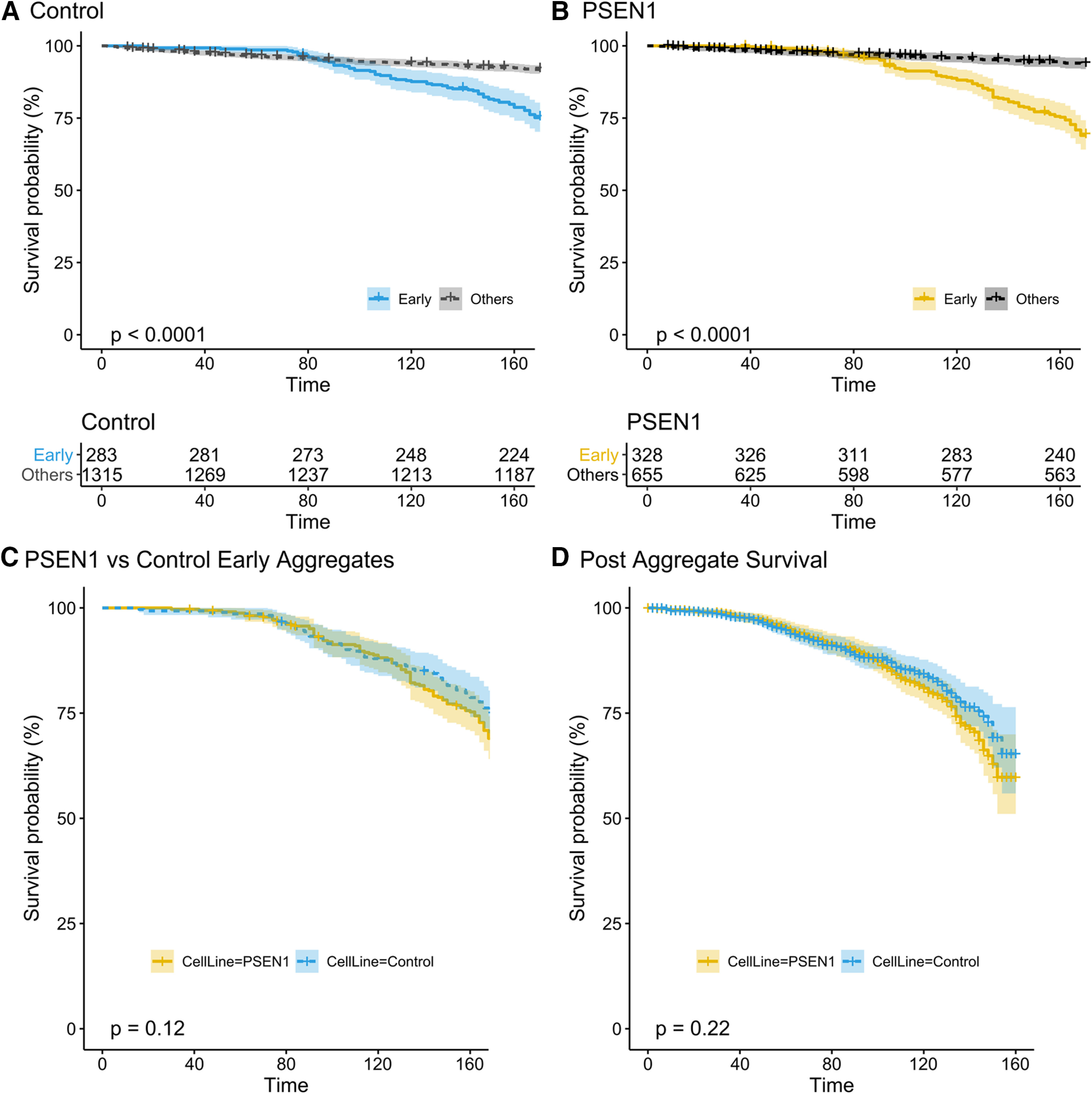
TauRD aggregate toxicity in control and familial AD iPSC neurons. ***A***, ***B***, Reduced survival in control (***A***) and PSEN1 L435F (***B***) neurons that form aggregates within 48 h after addition of seeds (Early) compared with all other neurons in the same well (Others), including cells that develop aggregates at later time points [*p* < 0.0001 for both (+) vs (–) aggregate comparisons; n.s. for line comparison by log-rank test; *n* = 4 independent neuronal differentiations; plots are ±95% confidence interval]. Crosses indicate censored cells. The number at risk for each time point is presented below. Time is represented in hours after seed addition. ***C***, Survival curves for early aggregate forming neuronal populations (<48 h after seed material addition) from control and PSEN1 mutant cells. ***D***, Survival curves for all aggregate-forming neurons from both cell lines beginning from the time of aggregate formation. Censor crosses are frequent in ***D*** because of censoring at the experimental end point, which occurs at a variable time with respect to aggregate formation in each cell (Extended Data Figures. 5-1, 5-2, 5-3).

The aggregate formation and neuronal survival data generated above was then used to produce Kaplan–Meier survival probability curves comparing neurons with and without aggregates under identical conditions in the same well. These data demonstrate that cell soma aggregate formation is associated with an increased risk of subsequent cell death. Since any TauRD aggregate-mediated neuronal death may be a time-dependent phenomenon, survival analysis was first performed on early aggregate-forming neurons, which would subsequently have the longest exposure to intracellular TauRD aggregates. In both control and PSEN1 cells, neurons that develop cell soma aggregates within 48 h of exposure to seed material are at a higher risk of subsequent death compared with all other neurons that have not yet developed aggregates at 48 h (*n* = 4, *p* < 0.0001; Fig. 5*A*,*B*). Similar results were also present for comparisons between the groups of neurons that developed aggregates at any time and those that remained aggregate free throughout the experiment (control, *p* = 0.041; PSEN1, *p* < 0.0001). These results indicate a deleterious effect of tau seed uptake followed by aggregation, as well as a delay between aggregation and death. At the time of cell death, fragmentation of the neuronal nucleus occurs along with lysis of the cell (Fig. 3*C–F*, Movies 1, 2, 3). Furthermore, there was a very weak positive correlation between TauRD fluorescence intensity and cell survival, present only in control cells, suggesting that the expression level of mutant TauRD was not a driving factor in neuronal cell death (control: *R*^2^ = 0.18, *p* = 0.044; PSEN1: *R*^2^ = 0.031, *p* = 0.72; Extended Data Fig. 5-1).

10.1523/JNEUROSCI.2590-20.2021.f5-1 Figure 5-1 TauRD reporter expression versus life span. Intensity of TauRD reporter expression shows a weak positive correlation with life span in control neurons. The above analysis is restricted to cells that died during the experiments and includes cells both with and without TauRD aggregation. Neuronal life span is plotted against normalized fluorescence intensity of the TauRD reporter per cell at the first imaging time point (t0). Pearson correlation is plotted for each cell line. *n* = 3 independent differentiations. In total, 131 control neurons and 135 PSEN1 mutant neurons are depicted. Normalized t0 values correspond to the average TauRD fluorescence intensity in an ROI centered over the tracked neuron divided by the average fluorescence intensity of all tracked neurons in each replicate, per cell line. Life span is represented in 2 h increments after seed addition. Download Figure 5-1, EPS file.

10.1523/JNEUROSCI.2590-20.2021.f5-2 Figure 5-2 TauRD aggregate toxicity in control and familial AD iPSC neurons. Survival curves for all neurons forming aggregates from control and PSEN1 mutant cell lines. Crosses indicate censored cells. Number at risk for each time point is presented below. Time is represented in 2 h increments after seed addition. Download Figure 5-2, EPS file.

10.1523/JNEUROSCI.2590-20.2021.f5-3 Figure 5-3 Cox proportional hazard model. Cox proportional hazard model comparing risk of cell death by cell line (PSEN1 mutant vs control) and time of cell soma aggregate formation (Early, before 48 h after addition of seed material). Lower values indicate a reduced risk of cell death compared with the reference group. Download Figure 5-3, EPS file.

Although the PSEN1 mutant iPSC neurons demonstrated accelerated aggregate formation, there was no difference in neuronal survival between the two cell lines after TauRD aggregation. Survival of control and PSEN1 early aggregate forming neurons was not significantly different (*p* = 0.12; Fig. 5*C*). As a group, PSEN1 neurons that developed aggregates at any time during the experiment did show a reduced survival compared with control neurons in the same category (*p* = 0.0038; Extended Data Fig. 5-2). However, a separate analysis of postaggregate survival time for all aggregate-forming neurons showed no significant difference between the control and PSEN1 cell lines (*p* = 0.22; Fig. 5*D*). These results suggest that reduced survival in the PSEN1 line on average is caused by accelerated aggregate formation and not a change in postaggregate survival. A Cox proportional hazard model supports this notion. While the hazard ratio for cell death was significantly lower in cells that had not developed aggregates by 48 h compared with those that had, there was no contribution of cell line identity to the hazard for cell death (Extended Data Fig. 5-3). These findings highlight the importance of using single-cell longitudinal analyses to decouple the rate of aggregate formation from subsequent neuronal phenotypes.

### Morphologic tau strains show differential toxicity and propagation

**Figure 6.**
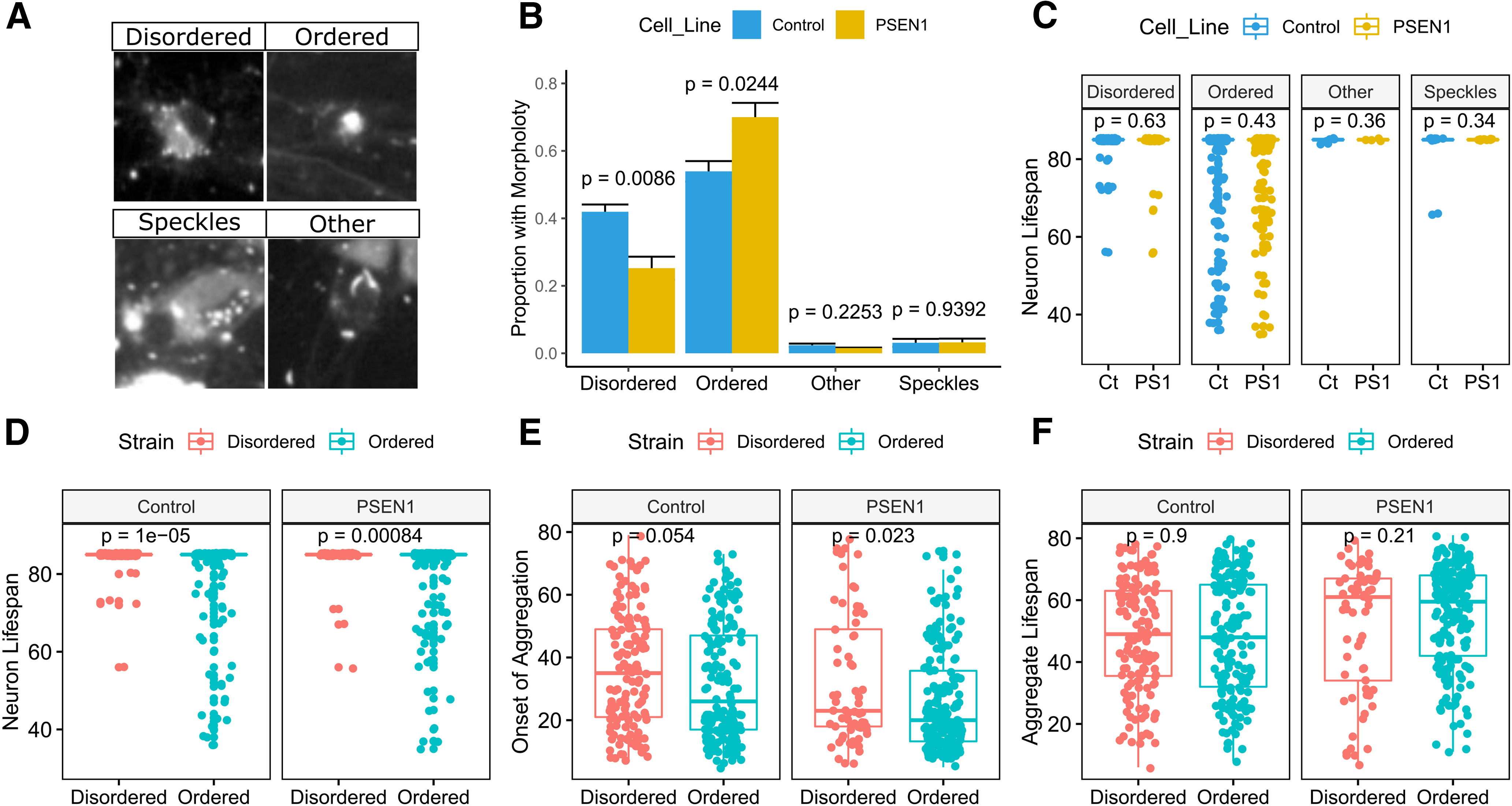
TauRD aggregate morphologies correlate with neurotoxicity. ***A***, Example images of observed TauRD aggregate morphologies (control neurons, *n* = 322; and PSEN1 L435F neurons, *n* = 267). ***B***, Relative proportions of each aggregate morphology for control and PSEN1 cell lines ±SE. ***C–F***, Boxplots. ***C***, Neuronal life span in control and PSEN1 iPSC neurons with aggregates, grouped by aggregate morphology (Ct, control, PS1, PSEN1). ***D–F***, Comparison of neuronal life span (***D***), Onset of aggregation (***E***), and aggregate life span (***F***), respectively, for cells with disordered and ordered aggregates, grouped by cell line. In ***B*** and ***C***, *p* values are for pairwise comparison between the two cell lines. In ***D–F***, *p* values are for pairwise comparison between aggregate types.

Prior studies indicate that multiple morphologic “strains” of tau can be identified and propagated as TauRD aggregates in HEK cells (Sanders et al., 2014). We assessed a random sample of tracked neurons across four biological replicates (*n* = 381 controls; *n* = 311 PSEN1 cells) and assigned strain morphologies where possible while blinded to cell line and survival status (control neurons, *n* = 322; PSEN1 neurons, *n* = 267). The following two main morphologic strains or patterns were present in iPSC neurons: (1) ordered aggregates, characterized by one or more large cytoplasmic condensations of TauRD, often with compaction over time; and (2) disordered aggregates, with many small cytoplasmic puncta that failed to coalesce during the imaging period (Fig. 6*A*). Rarely, iPSC neurons demonstrated prominent nuclear speckling of TauRD aggregates or a cytoplasmic aggregation pattern that did not fit into the above categories, sometimes characterized by a lenticular morphology (Fig. 6*A*). Surprisingly, the relative frequencies of ordered and disordered TauRD aggregates differed between the control and PSEN1 lines, with a shift toward more ordered aggregates in PSEN1 mutant neurons (between line frequency comparisons: disordered aggregates, *p* = 0.009; ordered aggregates, *p* = 0.024; Fig. 6*B*). Among the classes of aggregates identified, there were no cell line-dependent differences in neuronal life span (Fig. 6*C*). However, cells with ordered TauRD aggregates had a reduced life span compared with those with disordered aggregates (*p* = 1e-05 for control neurons and *p* = 0.0008 for PSEN1 neurons; Fig. 6*D*). This result suggests that aggregate strain morphology may play a role in tau toxicity or reflect a variable degree of exposure to toxic tau species. Interestingly, ordered TauRD aggregates began earlier during the course of experiments than those that were disordered (control neurons, *p* = 0.054; PSEN1 neurons, *p* = 0.023; Fig. 6*E*). Because it is possible that ordered aggregate morphologies represent the evolution of disordered aggregates over time, we assessed the amount of time that each aggregate was observed. There was no significant difference in this metric between the ordered and disordered morphologies (“aggregate life span”: control neurons, *p* = 0.9; PSEN1 neurons, *p* = 0.21; Fig. 6*F*). Thus, our results are consistent with strain differences in TauRD aggregates that represent an interaction between cell line and exogenous tau species and subsequently influence neuronal survival.

## Discussion

This work establishes real-time assessment of tau aggregation and subsequent neuronal survival in human iPSC neurons derived from patients with neurodegenerative disease. We have developed a live-imaging protocol that facilitates bulk quantification of TauRD reporter aggregation as well as longitudinal assessment of aggregation and neuronal death at single-cell resolution. Our implementation of a custom single-neuron tracking workflow results in a simple yet robust method for phenotyping individual cells throughout the course of experiments and allows censoring of neurons that leave the field of view or become otherwise obscured. Commercial equipment and an open source custom software simplify the acquisition and evaluation of the images and provide a robust technology for 4D analyses. These data are used to generate Kaplan–Meier survival probability curves comparing neurons with and without TauRD aggregates under identical conditions in the same well. We are then able to compare the survival of iPSC neurons derived from multiple patients, correcting for differences in the rate of TauRD aggregation.

We observed neurotoxicity following treatment with tau seeds in both control and PSEN1 L435F iPSC neurons. Neurons that aggregated the TauRD reporter were significantly more likely to undergo cell death during the course of the experiment compared with neurons in the same well that did not show TauRD aggregation. These results indicate a deleterious effect of tau uptake followed by reporter aggregation, as well as a delay between aggregation and death. To our knowledge, this is the first study to directly visualize tau aggregation and subsequent neuronal cell death from start to finish in single human neurons.

Neuronal death in our system is accelerated compared with that seen in human patients or *in vivo* in tangle-bearing MAPT mutant mouse models (de Calignon et al., 2010). Furthermore, acute toxicity was not present in mouse primary neurons treated with the seed-competent fraction of rTg4510 brain lysate (Takeda et al., 2015). This suggests that the observed rate of cell death in iPSC neurons may be increased by an interaction between soluble tau oligomer species and TauRD aggregation (Lasagna-Reeves et al., 2010; Ghag et al., 2018). Prior work of our group measuring tau toxicity in primary neurons was performed using population averages (Takeda et al., 2015). Applying longitudinal single-neuron survival analysis to this model may reveal more subtle tau-dependent neuronal cell death.

The absence of glia in these iPSC neuron cultures may also influence neuronal susceptibility to tau toxicity. Astrocytes, for instance, support the maturation and connectivity of neurons grown in culture (Molofsky et al., 2012; Nehme et al., 2018) and secrete neurotrophic factors that may have activity beyond those already present in the media formulation (Verkhratsky et al., 2016). However, astrocytes are a heterogeneous class of cells that can undergo reactive transformations, both *in vitro* and *in vivo*, and this may also influence their effects on neuronal survival (Roybon et al., 2013; Khakh and Sofroniew, 2015). Additionally, both astrocytes (Martini-Stoica et al., 2018; Perea et al., 2019; Kovacs, 2020) and microglia (Hopp et al., 2018) take up and metabolize tau in cell culture models and in some cases may release bioactive tau fragments back into the culture media. Studying the interplay between neurotrophic factor support and glial-dependent tau processing could be a fruitful avenue of further research approached using primary or iPSC-derived glia and a combination of coculture and conditioned media preparations.

In this study, survival in TauRD aggregate-bearing neurons is compared with adjacent neurons in the same dish as an attempt to control for overall exposure to potentially toxic soluble tau oligomers and other non-cell-autonomous factors. Prior work with rTg4510 brain lysates shows that uptake into neurons occurs within 24 h; well before the observed differences in survival between aggregate-bearing and aggregate-free neurons (Takeda et al., 2015). Still, it seems possible that cell-by-cell differences in the rate of tau uptake could contribute to variability seen in the onset of TauRD aggregation. However, experiments with labeled seed-containing brain lysates failed to establish a relationship between bulk lysate uptake and TauRD aggregation. Moreover, we found no correlation between TauRD expression and the onset of TauRD aggregation and a weak positive correlation between TauRD expression and survival in control iPSC neurons. These results suggest that tau seeding in iPSC neurons may be modulated by other homeostatic mechanisms and that these mechanisms may be activated at variable levels among adjacent neurons.

Important future directions will include elucidating the mechanisms of tau uptake and cell death in seed-containing iPSC neurons. Apoptotic neuronal death is believed to occur in AD and has been directly observed in animal models of AD and other tauopathies (Wirths and Bayer, 2010; Serrano-Pozo et al., 2011). However, the downstream effectors of neuronal tau toxicity *in vitro* are not well established. In addition, understanding the pathways involved in tau uptake is an essential component of future work, particularly in the context of recent reports identifying LRP1 as a ligand for tau internalization in human iPSCs (Evans et al., 2018; Rauch et al., 2020).

Our results show accelerated TauRD aggregation after seeding in iPSC neurons containing the PSEN1 L435F mutation compared with controls. An increased rate of TauRD reporter aggregation was present when measured both in the neurites and cell bodies of PSEN1 L435F iPSC neurons. Spontaneous tau aggregation is not a general feature of AD iPSC neuron models and was not seen in PSEN1 L435F iPSC neurons (Sproul, 2015; Mungenast et al., 2016; Oakley et al., 2020). However, there was no difference in postaggregate survival time between PSEN1 and control iPSC neurons, suggesting that the rate at which an aggregate forms is a kinetic choke point, which is possibly a time-limiting feature of tau-induced cytotoxicity. In accord with this interpretation, a Cox-proportional hazards model identified aggregation exposure time, but not PSEN1 mutation, as a significant contributor to overall neuronal survival.

These findings support a model where alterations in PSEN1 activity function upstream of tau aggregate formation in the pathogenesis of tau-induced neurotoxicity. In such a model, increased levels of longer Aβ species caused by mutations in PSEN1 would lead to increased tau phosphorylation which, in turn, would increase the rate of tau aggregation. Subsequent to tau aggregation, neuronal cell death would proceed by Aβ-independent mechanisms. Because this model posits that tau aggregation is the key insult that drives neuronal death, one would expect the post-tau aggregation rate of neuronal death to be equivalent between PSEN1 mutation-driven and sporadic causes of tau aggregation. This idea is consistent with numerous pieces of genetic and experimental data in Alzheimer's disease. Indeed, as is shown in our previous work as well as this publication, PSEN1 L435F iPSC neurons produce elevated levels of Aβ43 species and higher Aβ43/40 and Aβ42/40 ratios, indicating a potential cause of elevated seeding (Oakley et al., 2020).

We did not observe substantial incorporation of endogenous PHF-1 phosphorylated tau species into TauRD reporter aggregates. Qualitatively, this finding concurs with prior literature on TauRD aggregates, which have been shown to incorporate only small amounts of endogenous tau, sometimes apparent only by immuno-electron microscopy (Reilly et al., 2017). While endogenous tau is not necessary for propagation of misfolded tau species between neurons, it is thought to play a role in downstream neurotoxicity in mouse models (Wegmann et al., 2015). Additionally, the iPSC neurons used in this work express predominantly 3R tau isoforms at day 28 by Western blot (Oakley et al., 2020). This may influence the incorporation of endogenous tau into reporter aggregates, which are homologous to 4R tau.

In accord with prior studies using TauRD reporters in HEK cells, we identified several morphologic strains of aggregates that formed in response to tau seeds (Sanders et al., 2014; Kaufman et al., 2016, 2017). To our knowledge, this phenomenon has not yet been reported in human neurons. The specific TauRD aggregate morphologies identified in iPSCs are approximately equivalent to those seen in HEK cells and largely fell into categories of Ordered or Disordered. Interestingly, we found that Ordered aggregates were associated with reduced neuronal survival compared with Disordered aggregates and occurred more frequently in the PSEN1 L435F line than controls. These findings suggest that strain- and patient-specific differences in tau propagation could be studied using this approach. Because we used a homogeneous preparation from a transgenic animal as the source of tau seeds, our results suggest that multiple strains of seed-competent tau may exist contemporaneously in the same preparation, and potentially even in the same brain. Future work could assess whether specific morphologic strains of tau are present at differential rates in donated brain human tissues from different AD patients. Recent work from our laboratory and others demonstrates that there is a large degree of heterogeneity of the p-tau profile and tau seeding activity among AD patients (Holmes et al., 2014; Dujardin et al., 2020). It would be intriguing to ask whether this variability also translates into changes in tau aggregate morphology using a large panel of well characterized reference brain tissues.

The use of patient-derived iPSC lines as a source of human neurons will allow future exploration of patient-specific influences on neuronal tau seeding and survival. Given the wide range of Aβ plaque and tau tangle burden present in AD patients at autopsy (Braak et al., 2011; Kapasi et al., 2017), it will be informative to expand this technique across multiple sporadic and familial AD donors and to test the hypothesis that various genetic risk factors or specific phenotypes observed at autopsy reflect an endogenous propensity to tau aggregation in neuronal cells. Additionally, the ability to quantitively measure tau seed uptake and aggregation in human neurons should have utility in drug-screening and mechanistic studies focused on tau propagation in neurodegenerative disease.

Ultimately, this work demonstrates a tau seeding phenomenon in human iPSC neurons that is coupled with downstream neuronal death. Following seeding with brain lysates from mice overexpressing human P301L mutant tau, TauRD-YFP aggregation was associated with reduced neuronal survival in both control and PSEN1 L435 mutant iPSC neurons. TauRD reporter aggregation occurred more rapidly in the PSEN1 L435F line, in accord with our previous observations that these cells accumulate phospho-tau at baseline (Oakley et al., 2020). These findings were established using cell-by-cell comparison between neurons in the same well that did or did not aggregate the TauRD reporter. Furthermore, specific morphologies of TauRD aggregation were associated with increased neurotoxicity. The development of this methodology, along with the current results, suggests ways of investigating differences in tau toxicity in different tauopathies, and even among individual patients with sporadic AD.
